# Does left-handedness confer resistance to spatial bias?

**DOI:** 10.1038/srep09162

**Published:** 2015-03-17

**Authors:** Corinne A. Bareham, Tristan A. Bekinschtein, Sophie K. Scott, Tom Manly

**Affiliations:** 1 MRC Cognition and Brain Sciences Unit, 15 Chaucer Road, Cambridge, CB2 7EF, United Kingdom; 2 Queensland Brain Institute, The University of Queensland, Brisbane, Australia; 3 Department of Psychology, University of Cambridge, United Kingdom; 4 Institute of Cognitive Neuroscience, UCL, London, United Kingdom

## Abstract

We recently demonstrated that drowsiness, indexed using EEG, was associated with left-inattention in a group of 26 healthy right-handers. This has been linked to alertness-related modulation of spatial bias in left neglect patients and the greater persistence of left, compared with right, neglect following injury. Despite handedness being among the most overt aspects of human lateralization, studies of this healthy analogue of left neglect have only been conducted with predominantly or exclusively right-handed individuals. Here, with a group of 26 healthy non-right-handers we demonstrate that, unlike right-handers who showed a rightward shift in attention with drowsiness, non-right-handers showed the *opposite* pattern on an auditory spatial localization task. The current results are the first indication that factors linked to handedness can affect the development and extremity of spatial biases, potentially conferring resilience to clinical symptoms in non-right-handers and, given that 90% of us are right-handed, why *left* neglect is disproportionately persistent.

It has been argued that, in terms of our awareness of space, the two hemispheres of the brain have a competitive relationship, the right hemisphere pushing attention to the left, the left hemisphere pushing to the right^1^. The vulnerability of this finely balanced system to perturbation have been argued to account for remarkably high rates of lateralized inattention following unilateral stroke^2^. In the acute stage, up to 84% with right hemisphere stroke ignore information on the left and up to 65% of people with left hemisphere stroke ignore the right^3^. However, one of the most robust findings in neuropsychology is that recovery from this bias (termed Unilateral Spatial Neglect) is markedly asymmetric. Most cases of right neglect resolve relatively quickly and the overwhelming majority of patients showing chronic bias has right hemisphere damage and ignore the left^3^,^4^,^5^,^6^,^7^,^8^,^9^,^10^,^11^,^12^.

Influential accounts of this asymmetric recovery suggest that the intact right hemisphere, specialized in spatial function, can compensate for a damaged left hemisphere whilst the left hemisphere is ill equipped to reciprocate after right hemisphere damage^2^. Mounting evidence from human functional brain imaging that relevant spatial networks have a bilateral distribution has, however, challenged this view^13^. Based on the anatomy of spatial neglect and the observed modulatory influence of alertness on spatial function, it has been proposed that damage to a right hemisphere lateralized Ventral Attention Network (VAN) implicated in alertness, may account for the disproportionate maintenance of left neglect^13^.

Left-handers make up about 10% of the population. A consistent finding has been that left-handers show a less extreme, more mixed hand/foot preference and, consequently, the term ‘non-right-handed’ is sometimes used and adopted here. Handedness is one of the most obvious human lateralizations of function and yet surprisingly little is known about how it may interact with spatial neglect. Neuroscience studies, excepting those concerned with handedness, often exclude non-right-handers in order to see the ‘normal’ pattern. In major studies on the incidence and recovery from left neglect, non-right-handed patients are explicitly excluded^4^,^5^,^6^, handedness is not reported^3^,^7^,^8^,^9^,^10^ or severity and pattern of neglect is not reported separately^11^,^12^. Until relatively recently there were not strong grounds to expect marked differences. The majority of non-right-handers (75%) and right-handers (95%) show left hemisphere lateralization of language^14^ and spatial memory is predominantly right hemisphere lateralized in approximately 75% of right- and non-right-handers^15^. Studies that have compared lateralization of handedness, language and spatial function in the same population suggest that they are not manifestations of a common underlying process but occur probabilistically and independently^15^. Of four brain networks recently reported to show reliable lateralization across a large group of participants, only one showed a significant interaction with handedness^16^. In right-handers the VAN, discussed above, was right hemisphere lateralized. In non-right-handers it was bilateral or even slightly left-lateralized. This intriguing finding suggests that another factor behind non-right-handers' absence from the extensive spatial neglect literature maybe that they are not meeting studies' inclusion criteria, in other words, their bilateral distribution of the relevant network confers resilience to persistent spatial effects of unilateral lesions. The corollary is that one factor contributing to the disproportionate persistence of *left* neglect may be that 90% of us are right-handed.

To fully examine this, necessarily large longitudinal stroke studies would be required to recruit sufficient non-right-handers. An initial indication of its likelihood is offered, however, by a phenomenon that has been linked with VAN modulation of spatial function in the general population^13^. Healthy participants (nearly all right-handed) in states of low alertness show relative inattention to the left^17^,^18^,^19^,^20^,^21^. Most recently we have demonstrated a marked tendency in right-handers to mislocate left tones to the right as they transition towards sleep^17^, suggesting convergence between left neglect and the effects of drowsiness on healthy spatial bias. Here, 26 non-right-handed volunteers (who self-identified as ‘left-handed’) underwent the identical experimental procedure. Participants reclined with their eyes closed and were asked to judge whether each of a series of tones (lateralized up to 60° left and right of midline) occurred to the left or right. EEG was recorded throughout the task. As alertness declines, increases in EEG theta and decreases in alpha activity are observed^22^ particularly in certain sub-bands^23^,^24^. Accordingly, to track alertness the ratio of theta (sub-band 4–6 Hz) and alpha (sub-band 10–12 Hz) was automatically calculated over the 4 seconds before each tone. Trials in the upper and lower quartiles of each participant's lower theta-upper alpha ratio distributions were categorized as relatively drowsy and alert respectively.

## Results

**Figure 1:**
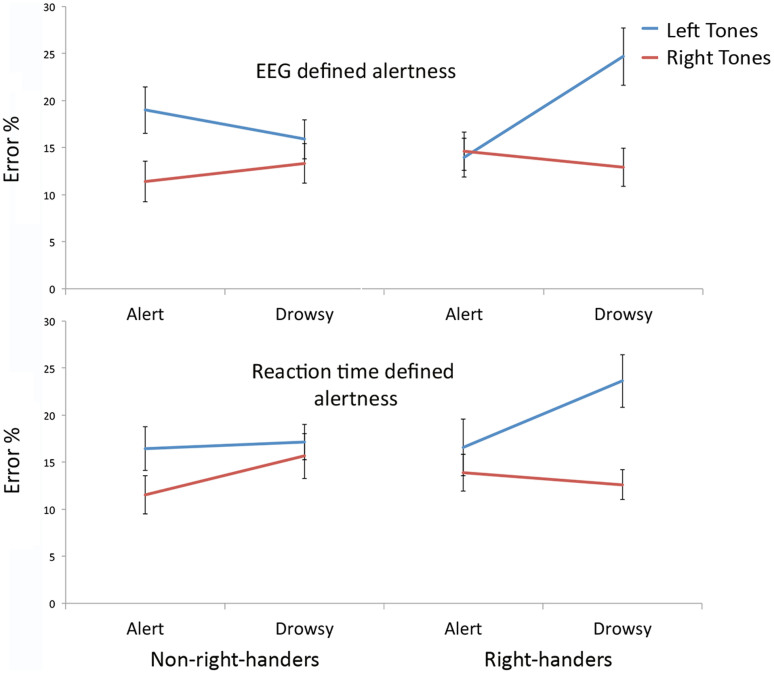
Error rates for non-right-handed and right-handed participants in detecting tones presented to the left or right, under conditions of relatively high or low alertness, whether alertness is defined using EEG (top) or behaviourally from reaction times (bottom). Non-right-handers show no hint of the marked increased tendency of right-handers to report left tones as right when drowsy.

As was previously reported for right-handers^17^, there was a statistically significant alertness x side-of-tone interaction (*F*(1,25) = 4.30, *p* = 0.049, Cohen's *d* = 0.83) in non-right-handers' performance. However, it was in the *reverse* direction. As shown in Figure 1, whilst right-handers' error rates on left-tones increased from 13.94% (*SD* = 10.49) to 24.67% (*SD* = 15.48) from relatively alert to relatively drowsy periods, non-right-handers' left-tone error rates in the same contrast *declined* from 18.98% (*SD* = 12.53) to 15.90% (*SD* = 10.63). Right-handers' error rates on right-tones decreased from 14.61% (*SD* = 10.53) during alert performance to 12.92% (*SD* = 10.35) when drowsy. In contrast, non-right-handers' right-tone error rates increased from 11.40% (*SD* = 11.08) to 13.34% (*SD* = 10.78) in the same comparison. When the handedness groups were formally compared using data from Ref. 17 a robust handedness x alertness x side-of-tone interaction on error rates was observed (*F*(1,50) = 20.75, *p* < 0.001; Cohen's *d* = 1.29). Whilst hand preference had a strong effect, neither the hand used to make the responses nor the sex of participants interacted with the key alertness x side-of-tone or handedness x alertness x side-of-tone effects (see supplementary results for details and Signal Detection Theory – SDT – analyses).

Sleep onset is linked with generally increased response latency and also variability^22^. A second, independent behavioral index of alertness was therefore derived based on participants' reaction time and response time variability (see supplementary materials). Unlike the right-handers, whose trials were divided into alert and drowsy using the same index, the non-right-handed participants showed no response time defined alertness x side-of-tone interaction in error rates (*F*(1,25) = 1.00, *p* = 0.33) and, when the two groups were formally compared, there was again a robust handedness x alertness x side-of-tone interaction (*F*(1,50) = 6.40, *p* = 0.015, Cohen's *d* = 0.71; see supplementary materials for details and SDT analyses).

**Figure 2:**
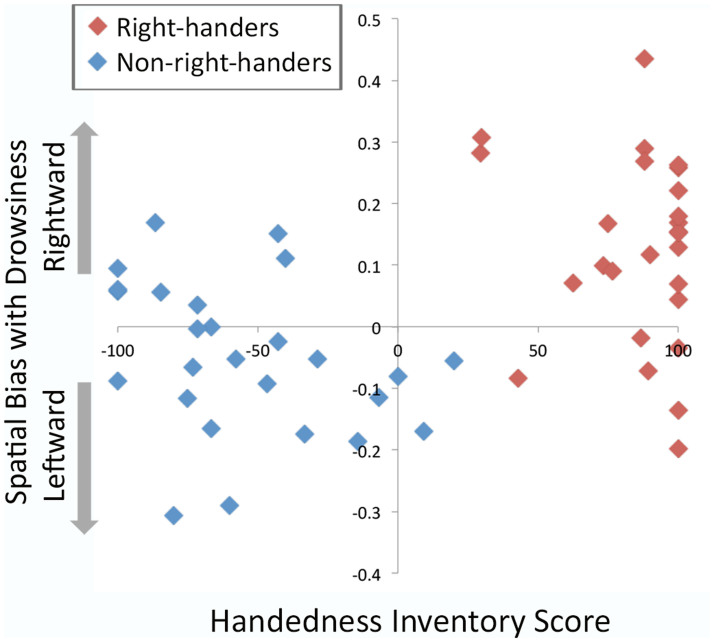
Individuals' Oldfield Handedness Questionnaire Scores plotted against Drowsiness Bias Score ((Drowsy left error % - Alert left error %) – (Drowsy right error % - Alert right error%)). Right-handers have more consistent handedness scores and typically a rightward drowsiness bias on the task whilst non-right-handers have less consistency in handedness scores and are more likely to show a leftward drowsiness bias.

The handedness groups did not differ in age (*F*(1,50) = 0.08, *p* = 0.93) or sex ratio (*Chi^2^* = 1.95, *p* = 0.16). The consistency of hand-preference was measured by the Edinburgh Handedness Inventory^25^ that gives scores between 100 (consistently right-handed preference across tasks) and −100 (consistently left). As is often reported, the non-right-handed sample were notably less consistent in their preference (−56.83, *SD* = 31.10 vs. 85.37, *SD* = 21.64; *F*(1,50) = 14.74, *p* < 0.0001, sign ignored). However, the consistency of hand preference was uncorrelated with the degree of change between alert and drowsy trials for either group (see Figure 2 and supplementary materials). A clinical electrophysiologist categorized all EEG traces according to the modified version of the Hori Scale^26^ of sleep stages. In terms of their propensity to drowsiness and their actual levels of drowsiness during the experiment, the non-right-handers did not differ from the previously reported right-handers in terms of Hori mean or maximum scores (*F*(1,50) = 0.69, *p = * 0.41; *F*(1,50) = 0.21, *p = * 0.65), mean or maximum theta:alpha scores (*F*(1,50) = 0.62, *p* = 0.44; *F*(1,50) = 0.23, *p = * 0.63), reaction times or variability in reaction times (*F*(1,50) = 0.94, *p = * 0.34; *F*(1,50) = 1.91, *p = * 0.17), proportion of missed responses (*F*(1,50) = 0.14, *p = * 0.71), or their self-reported ease of sleep onset in everyday life (Epworth Sleep Scale^27^ *F*(1,50) = 1.5, *p* = 0.23, see supplementary materials). In other words, we can be confident that the difference in error patterns is not related to non-right-handers becoming less drowsy than the previously reported right-handed group.

## Discussion

It has been reported that the Ventral Attention Network (VAN) is predominantly right hemisphere lateralized in right-handers but bilateral or even slightly left-lateralized in non-right-handers^16^. As discussed, damage to this network has been implicated in the etiology of unilateral spatial neglect, in the disproportionate persistence of left compared with right neglect and the emergence of left-neglect like patterns in healthy participants in states of low alertness^13^. An interesting and novel test of the latter is to examine whether non-right handed people show similar alertness based changes in spatial attention. Here we have indeed observed a very different pattern of changes in spatial awareness as participants transitioned from relatively alert to relatively drowsy states. The markedly increased tendency of drowsy right-handers to report left-tones as ‘right’ was entirely absent, or reversed in the non-right-handed group. By extrapolation, non-right-handers may have advantages in overcoming the impact of right hemisphere lesion on spatial function via more bilateral, or even perhaps more left-lateralized VAN organization. In addition to being underestimated in the left unilateral spatial neglect literature due to relative rarity and exclusion, non-right-handers may have resilience to persistent left spatial neglect and therefore simply not be included in relevant studies. In contrast, right-handers who suffer right hemisphere damage and who are right-lateralized for relevant spatial functions *and* VAN, may be caught in a ‘perfect storm’ in which multiple factors align to perpetuate left neglect. The issue of whether non-right-handers show a reverse pattern (as suggested by our theta:alpha analysis) or simply the absence of a lateralized pattern of change with drowsiness (as suggested by our RT analysis) is important. A small clinical study may be of relevance^28^. This reported, as expected, that recovery from *right* unilateral spatial neglect was generally rapid for right-handers. However, 8/9 non-right-handers showed persistent right unilateral spatial neglect. More research is required on recovery of right- and non-right-handers from right and left spatial neglect. Whilst they may be understudied due to their relative rarity, by inference from the current study, non-right handed people may have much to tell us about the nature of, and recovery from, neglect.

Handedness is thought to be determined by multiple genetic loci^29^ and there are debates about whether it is best considered as a categorical or continuous variable, and how it is best assessed. It might be expected, given the overall difference between self-reported right- and non-right-handed groups presented here, that the consistency of hand preference could influence the magnitude and direction of the change between drowsy and alert periods. The lack of a significant correlation in the right-handers in this respect may be attributable to the relatively high proportion with maximum handedness scores limiting variability. For the non-right-handers however, as is commonly reported, there was a much greater range of scores (including two who reported using their right hands for more tasks than their left) and yet no significant correlation with change in bias was observed. One possibility is that the effect is related to a factor that correlates with right vs. more mixed handedness (such as VAN distribution) rather than which hand is preferred for certain tasks (which may in part be determined by learning/cultural/affordance pressures, and is the basis for the consistency of hand-preference measure used in this study). Against this it is possible that the much greater shift in performance seen in right-handers is related to the more strongly lateralized preference in this group. Further research that is better powered to detect such relationships and which employ a wider range of handedness measures is required. There are also reports in the neglect literature of unimanual left-hand movements increasing awareness of left stimuli (at least in right-handers) suggestive of more direct links between motor activity and lateralized attention^30^. If it is the case that non-right-handers' heightened level of activity and experience of using the left-hand is important in offsetting rightward attentional bias this would have clear implications for rehabilitation strategies in the case of neglect.

As well as the clinical implications, changes in the perception of stimuli with sleep onset are of interest in understanding differences between conscious and unconscious processes. Previous research indicates that prefrontal cortical activity, implicated in conscious awareness, is reduced during sleep whilst activity in sensory areas in response to a stimulus may be relatively unchanged^31^. In line with this, sleeping research participants have been shown to be able to continue a practiced discrimination response to stimuli but unable to remap responses in a goal directed manner^32^. Falling asleep is a process that takes several minutes during which, as the current study suggests, conscious perception of different aspects of the stimulus (such as location) may be differentially affected whilst basic detection continues. Given recent results^32^ it would it would be of interest to examine whether right- and non-right-handed sleeping participants could continue to discriminate left and right tones and the resulting relative error rates.

## Methods

Ethical approval for the study was given by the Cambridge Psychology Research Ethics Committee (CPREC 2009.69). Written informed consent was obtained before any testing commenced.

### Participants

Twenty-six self-reported non-right-handed healthy adults aged 18–35 (mean 24.44 years, *SD* 4.48, 12 women) with self-reported normal hearing participated. Mean Handedness score on the self-report Edinburgh Handedness Inventory was −54.60 (SD 35.03).

### Design

The task required participants to indicate after each of a series of lateralized auditory stimuli whether this had occurred to their right or left side. Piloting suggested that ceiling level performance on stimuli lateralized >60° of midpoint. Accordingly the final stimulus set comprised 324 tones lateralized between 1.86° and 60° to the left and right. In the participants' task, each trial began with a wait of between 5–8 seconds (selected at random) before each stimulus. On the basis that the exclusive presentation of difficult-to-judge stimuli might encourage participants to simply guess, the relatively easy-to-judge (and therefore likely less sensitive) 40–60° stimuli were used sparingly, each being presented just once during the task. The remaining stimuli were each presented 6 times with, aside from this weighting, stimuli selection being random for each trial for each participant. The task was scripted using MATLAB and delivered via a Dell laptop.

### Procedure

All methods were conducted in accordance with the CPREC guidelines. After providing informed written consent, participants filled out the Epworth Sleepiness Scale^27^ and the Oldfield (Edinburgh) Handedness Questionnaire^25^ and were then fitted with a 129-channel electrolyte cap (EGI systems) to record EEG throughout the session. Participants were individually tested in a quiet, dark room in a comfortable reclined chair. Responses were made via a button box resting centrally on the abdomen. To control for any factors related to lateralized movements, 9 participants (5 female) were randomly allocated to respond via the button box with the thumbs from both hands, 8 (4 female) to respond with the middle and index finger of the left hand, and 9 (2 female) with the middle and index fingers of the right hand. To minimize potential confusion about responses and encourage likely drowsiness through minimizing the cognitive demands of the task, the left and right buttons always served to signal left and right responses respectively. Participants were asked to keep their eyes closed throughout and not worry if they felt drowsy or fell asleep. They were told that if they did fall asleep, as indicated by 3 consecutive missed responses, the examiner would increase the volume of the stimuli until responding recommenced. If no response was detected, the program automatically triggered the next trial after a delay of 5 seconds from tone onset. Reaction time (RT), omissions and accuracy were recorded. Total testing time varied depending on RT and omissions but was typically around 40–50 minutes.

### EEG analysis

Upper alpha: lower theta ratios from the 129 electrodes were computed over the 4 s preceding each tone. Lower alpha was defined as 10–12 Hz and upper theta as 4–6 Hz^17^,^23^,^24^. A PCA was used to reduce the data to one vector, and the first principal component was taken as the ratio for that trial. Each trial for each participant was then categorized as relatively drowsy (top 25% of lower theta-upper alpha ratio scores) or alert (lowest 25%). The electrophysiologist who had previously scored right-handers'^17^ EEG traces on the Hori scale also scored the non-right-handers EEG in the current study, using the 10–20 system blind to all behavioural data and the results of the upper theta: lower alpha ratio generation. The wake stages are identified by the presence of alpha (H1-2), with relaxed wakefulness indicated by alpha suppression (H3), and drowsiness determined by alpha flattening (H4), ripples (H5), vertex sharp waves humps (H6), trains of humps (H7), humps with incomplete spindles (H8), and deeper sleep determined by the presence of spindles or K-complexes (H9)^26^.

### Reaction Time Analysis

Trials were defined as alert or drowsy using a coefficient of variation method. The coefficient of variation is a method for examining variability that takes into account the overall magnitude (standard deviation/mean). See supplementary methods for details.

## Author Contributions

C.A.B., T.M., S.K.S. and T.A.B. designed the research; C.A.B. and T.A.B. performed the research; C.A.B., T.M. and T.A.B. analyzed the data; and C.A.B., T.M. and T.A.B. wrote the manuscript and supplementary information.

## Supplementary Material

Supplementary Information Supplementary Information
