# Supplementary material for: Does left-handedness confer resistance to spatial bias?
Source: Sci Rep. 2015 Mar 17;5:9162. doi: 10.1038/srep09162 (PMC4361991; doi:10.1038/srep09162)
Supplement: Supplementary Information [file srep09162-s1.doc]

Does left-handedness confer resistance to spatial bias?

Corinne A. Bareham^1,2^, Tristan A. Bekinschtein^1,3^, Sophie K. Scott^4^ & Tom Manly¹

¹ MRC Cognition and Brain Sciences Unit, 15 Chaucer Road, Cambridge, CB2 7EF, United Kingdom

^2^Queensland Brain Institute, The University of Queensland, Brisbane, Australia.

^3^ Department of Psychology, University of Cambridge, United Kingdom

^4^ Institute of Cognitive Neuroscience, UCL, London, United Kingdom

Running Title: Left-handers sleep onset and spatial attention

Corresponding author: Bareham, C. A.

Address: The Queensland Brain Institute, The University of Queensland, St Lucia QLD 4072, Australia.

Telephone: 01223 355294

Email: [corinne.bareham@gmail.com](mailto:corinne.bareham@mrc-cbu.cam.ac.uk)

Category: BIOLOGICAL SCIENCES – Psychological and Cognitive Sciences

Key words. Spatial Attention, Sleep, arousal, handedness.

**Supplementary Methods**

*Stimuli*

The stimuli were created by playing a recording of a single guitar chord at constant volume and elevation (1m) at points within a 180°, 2m radius semicircle around a seated experimenter looking straight ahead and wearing in-ear microphones. A 400 ms guitar chord (D centred around 146.382 Hz) was used rather than pure tone both because of its relaxing harmonic qualities and because wider bandwidth sounds are easier to localize. Stereo in-ear microphone recordings capture complex cues for localization including volume differences due to distance and head shadow, the effect of the pinna, room reverberations and so on, allowing the scene to be realistically recreated when heard through in-ear earphones. During stimulus creation, 19 presentations were made at approximately 4° increments starting from 90° left/right locations heading towards midline. The remaining six increments from 12º-2º lateralization narrowed to approximately 2º to create more instances of near midline stimuli. Finally, two midpoint stimuli were included (one as approached from the right and one from the left), making a total of 25 left-sided, 25 right-sided and two midpoint stimuli. All sound files were then duplicated and stereo-flipped in Audition© software, creating a further 52 stimuli such that any extraneous noises, room cues etc. appeared equally in left and right-stimuli sets. Midpoint stimuli appeared 6 times but were excluded from further analysis because no response could be considered an error.

*EEG Pre-processing*

EEG data were filtered at 0.5 – 40Hz band pass and segmented -4000ms to +4ms relative to stimulus presentation. The remaining preprocessing was conducted offline in MATLAB using EEGLAB. Data were re-referenced to the average of 129 channels. Bad channels (due to movements, blinks etc.) were identified by extreme filtering at 150 and 150µv and visual inspection, with spherical spline interpolation being applied based on the voltages of neighbouring electrodes. No more than 5 channels were interpolated per participant (where > 5, those segments were rejected). Data from the first 10 trials were excluded as practice.

*Reaction Time Analysis*

Trials were defined as relatively alert or drowsy based on participants’ response times as follows. For each participant, a moving average of reaction times (correct and incorrect) was calculated over the previous 10 trials with these local values then being averaged to form a grand mean. Each trial, being represented by its own and the mean of the previous 9 reaction times, was then categorized as above (‘drowsy’) or below (‘alert’) each participant’s grand mean. For each rolling average over the current and previous 10 trials, a coefficient of variation was also calculated. The coefficient of variation is a method for examining variability that takes into account the overall magnitude (standard deviation/mean). A grand mean of all the coefficients of variation was calculated for each participant and then each trial categorized as ‘drowsy’ or ‘alert’ depending on whether its smoothed value fell above or below this mean. This decoupling of response time variability and overall response times meant that some trials would be categorized as, say, ‘drowsy’ according to the generally inflated reaction times over it and the last 9 trials but as ‘alert’ according to the relatively low level of variability in those reaction times. To best capture periods of most likely ‘drowsy’ or ‘alert’ responding we therefore selected trials that were congruent on RT *and* variability definitions for each category. It should be noted that the rolling average method effectively removes any confound of task difficulty (e.g. RTs being long because a difficult to judge tone close to midline has been presented). Within the random presentation order, trials of different levels of difficulty will have been presented during the 10 trials contributing to the rolling average.

**Supplementary results**

*Theta:alpha defined alertness x side of tone interaction in non-right-handers.*

The first result reported in the main paper is a statistically significant interaction between theta:alpha defined alertness (upper vs. lower quartile) and side-of-tone (left vs. right). In detail, the repeated measures ANOVA revealed no overall effect of alertness on error rates (error rate during alert trials 15.19%, *SD* = 12.32, drowsy 14.62%, *SD* = 10.68; *F*(1,25) = 0.13, *p* = 0.72) nor of side-of-tone (error rate on left tones 17.44%, *SD* = 11.60, right-tones 12.37%, *SD* = 10.87; *F*(1,25) = 2.06, *p* = 0.16), just statistically significant alertness x side-of-tone interaction (*F*(1,29) = 4.30, *p* = 0.049).

Relative error rates on left- and right-tones can be expressed as single measures within the Signal Detection Theory (SDT) framework. Accordingly *d’* (sensitivity) was calculated using ϕ-1(H) - ϕ-1(F) and *c* (criterion) using (ϕ-1(H) + ϕ-1(F))/2 where H was the proportion of left-stimulus trials with a correct response and F was the proportion of right-stimulus trials with a left response. The *d’* and *c* values were entered into separate repeated measures ANOVAs with the within-subject factors of alertness (trials from the lower quartile of each participant’s theta:alpha distribution vs. trials from the upper quartile). There was no statistically significant effect of alertness on sensitivity (*d’* alert = 2.42, SD 0.87; drowsy 2.33, *SD* = 1.30; *F*(1,25) = 0.093, *p* = 0.76) or criterion (*c* alert 0.35, *SD* = 0.76; drowsy 0.39, *SD* = 0.76; *F*(1,25) = 0.10, *p* = 0.76).

*Handedness x theta:alpha defined alertness x side-of-tone interaction.*

The second result reported in the main paper was a significant 3-way interaction between handedness (right vs. non-right-handed), theta:alpha defined alertness (upper vs. lower quartile) and side-of-tone (left vs. right) *F*(1,50) = 20.75, *p* < 0.001; Cohen’s *d* = 1.29). The results of this repeated-measures ANOVA in detail were as follows. There was no overall effect of alertness (alert error rate 14.73%, *SD* = 11.36; drowsy 16.71%, 12.74; *F*(1,50) = 2.53, *p =* 0.12). There was a significant overall effect of side-of-tone (error rate left-tones 18.37%, *SD* = 12.92; right-tones 13.07%, *SD* = 10.59; *F*(1,50) = 4.91, *p* = 0.03). There was a statistically significant interaction between handedness and alertness in the sense that overall error rates changed differentially between the groups over relatively alert and drowsy trials (non-right-handers alert error rate 15.19%, *SD* = 12.32; drowsy 14.62%; right-handers alert error rate 14.28%, *SD* = 10.41; drowsy 18.79%, *SD* = 14.32; *F*(1,50) = 4.19, *p =* 0.046). There was no significant interaction between handedness and the relative rates of errors for left- and right-tones in general (non-right-handers error rate on left-tones 17.44%, *SD* = 11.60; right-tones 12.37%, *SD* = 11.60; right-handers error rate on left-tones 19.30%, *SD* = 14.17; right-tones 13.77%, *SD* = 10.37; *F*(1,50) = 0.01, *p* = 0.92). There was an overall trend towards a significant interaction between alertness and side-of-tone driven by the steep increase in left-tone errors by right-handers when drowsy (alert errors on left-tones 16.46%, *SD* = 11.72; right-tones 13.00%, *SD* = 10.82; drowsy errors on left-tones 20.28%, *SD* = 13.87; right-tones 13.13%, *SD* = 10.46; *F*(1,50) = 3.74; *p* = 0.059).

As above, the relative error rates on left- and right-tones of the two handedness groups during theta:alpha defined alert and drowsy trials can be expressed in terms of SDT measures. These were subjected to separate repeated measures ANOVAs with the within-subject factor of alertness (high vs. low) and the between-subject factor of handedness. There was no significant interaction between alertness and handedness on *d’* (*F*(1,50) = 2.52, *p =* 0.12) but a significant alertness x handedness interaction was observed on *c* (*F*(1,50) = 5.38, *p =* 0.025). For right-handers, *c* changed from 0.031 (*SD* = 0.58) on relatively alert trials to 0.49 (*SD* = 0.65) on relatively drowsy trials whilst for non-right-handers the values were more stable (alert 0.35, *SD* = 0.76; drowsy 0.39, *SD* = 0.76).

*The effect of sex and hand-of-response*

The above ANOVAs were repeated with sex and hand-of-response (both vs. left vs. right) as between-subject factors. The results indicated that neither hand-of-response nor sex significantly interacted with the theta:alpha defined alertness x side-of-tone interaction in the non-left handers considered in isolation (gender *F*(1,24) =0.06, *p* = 0.81; hand-of-response *F*(2,23) = 0.60, *p* = 0.56) or with the handedness x alertness x side-of-tone interaction across both handedness groups (sex *F*(1,48) = 1.72, *p* = 0.20; hand-of-response *F*(2,46) = 0.61, *p* = 0.55).

*Correlations between consistency of hand preference and change in performance from theta:alpha defined alert to drowsy trials*

Pearson correlations were performed between variables reflecting change in error rates for left and right-tones (both separately and combined within SDT measures) between theta:alpha defined alert and drowsy trials and the consistency of hand preference as measured by the Edinburgh Handedness Inventory. The results, which showed no significant relationships for either non-right-handers or right-handers, are presented in table 1.

Supp. Table 1

|  | drowsy – alert left-tone error rate | drowsy – alert right-tone error rate | drowsy – alert *d’* | drowsy – alert *c* |
| --- | --- | --- | --- | --- |
| non-right-handers | -0.1, *p* = 0.63 | -0.31, *p* = 0.13 | -0.07, *p* = 0.74 | 0.02, *p* = 0.93 |
| right-handers | -0.22, *p = 0.28* | 0.09, *p* = 0.68 | -0.2, *p* = 0.33 | -0.21, *p* = 0.30 |

Supp. Table 1. Pearson correlation coefficients (and p values) between Oldfield (Edinburgh) Handedness Inventory Scores and measures of change with drowsiness for non-right-handed and right-handed participants.

*Comparison of non-right-handers and right-handers’ theta:alpha, RT, omissions and Hori Scale Scores.*

In the main paper the results of a series on one-way ANOVAs showing that non-right-handers did not differ from previously reported right-handers in important respects. The results in detail were as follows:

Supp. Table 2.

| Variable | Non-right-handers mean (SD) | Right-handers mean (SD) | ANOVA |
| --- | --- | --- | --- |
| Mean theta:alpha score | 0.01 (0.33) | 0.16 (0.16) | *F*(1,50) = 0.62, *p* = 0.44 |
| Maximum theta:alpha score | 23.56 (15.25) | 21.69 (12.59) | *F*(1,50) = 0.23, *p =* 0.63 |
| Mean correct reaction time (ms) | 1073.32 (318.20) | 1172.74 (414.99) | *F*(1,50) = 0.94, *p =* 0.34 |
| Correct reaction time variability (standard deviation) | 543.28 (228.06) | 646.27 (302.83) | *F*(1,50) = 1.91, *p =* 0.17 |
| Mean Hori Score | 2.66 (1.04) | 2.89 (0.98) | *F*(1,50) = 0.69, *p =*0.41 |
| Maximum Hori Score | 6.15 (2.65) | 6.46 (2.18 | *F*(1,50) = 0.21, *p =* 0.65 |
| Proportion of trials with absent responses | 0.061 (0.09) | 0.070 (0.07) | *F*(1,50) = 0.14, *p =* 0.71 |

*RT defined alertness x side-of-tone and handedness x alertness x side-of-tone*

As reported in the main paper, there was no RT defined alertness x side-of-tone interaction in the non-right-handers but a handedness x alertness x side-of-tone interaction when both groups were examined. In detail, a repeated measures ANOVA was conducted on error rates for non-right-handers with the within-subject factors of RT defined alertness (upper vs. lower interquartile range of RT index) and side-of-tone (left vs. right). This revealed no main effect of alertness (*F*(1,25) = 3.46, *p* = 0.073), no main effect of side-of-tone (*F*(1,25) = 0.91, *p* = 0.35) and no alertness x side-of-tone interaction (*F*(1,25) = 1.00, *p* = 0.33). When the same analysis was conducted across both handedness groups with the additional between-subjects factor of handedness it revealed a statistically significant main effect of alertness (*F*(1,50) = 6.17, *p* = 0.02), a near significant effect of side-of-tone (*F*(1,50) = 4.34, *p* = 0.042), no significant alertness x side-of-tone interaction, (*F*(1,50) = 1.05, *p* = 0.31), no significant handedness x alertness interaction (*F*(1,50) = 0.03, *p* = 0.85), no handedness x side-of-tone interaction (*F*(1,50) = 0.60, *p* = 0.44) but a statistically significant handedness x alertness x side-of-tone interaction (*F*(1,50) = 6.40, *p* = 0.015, Cohen’s *d*  = 0.71). Whilst right-handers error rates on left-tones increased from RT defined alert to drowsy trials (from 16.75%, *SD* = 15.38, to 23.62%, *SD* = 14.19), in non-right-handers this was more stable (from 16.44%, *SD* = 11.97, to 17.11%, *SD* = 9.54). On right-tones, right-handers’ error rates declined slightly from alert to drowsy trials (from 13.88%, *SD* = 10.13 to 12.59%, *SD* = 8.08) whilst non-right-handers’ showed a modest increase (from 11.52%, *SD* = 10.26 to 15.65%, *SD* = 12.15).

These results can be represented within a SDT framework using *d’* and *c* as calculated above. Repeated measures ANOVA on the dependent of *d’* and the factor of RT defined alertness (high vs. low) revealed a statistically significant change in non-right-handers between alert (2.86, *SD* = 0.98) and drowsy trials (1.84, *SD* = 0.73; *F*(1,25) = 30.15, *p* < 0.001*,* Cohen’s *d*  = 2.2). However, when both handedness groups were considered there was no significant interaction between handedness and change in *d’* (*F*(1,50) = 0.27, *p* = 0.61). The same analyses were performed for *c.* This indicated that, for non-right-handers there was no significant change in *c* between RT defined alert and drowsy trials (0.22, *SD* = 0.85 and 0.24, *SD* = 0.59 respectively; *F*(1,25) = 0.03, *p* = 0.86.) There was however a statistically significant handedness x alertness interaction on *c* when both groups were considered in the same ANOVA (*F*(1,50) = 6.46, *p* = 0.014, Cohen’s *d* = 0.79). Unlike non-right-handers, right-handers’ *c* values changed from alert (-0.08, *SD =* 0.89) to drowsy trials (0.45, *SD =* 0.38).

*Hori and omissions defined alertness x side-of-tone and handedness x alertness x side-of-tone analyses.*

In the previous report on right-handed participants we included analyses of left- and right-tone error rates during epochs of task performance characterized as alert or drowsy according to two further independent measures of alertness. The first was based on an electrophysiologists’ independent scoring of participants’ EEG traces according to the Hori scale and the second a comparison of blocks with 2 or more omissions (suggestive of sleep onset) and blocks with no omissions. In terms of the Hori scale conventional categorization of trials with scores > 3 as drowsy and 1-3 as alert indicated no alertness x side-of-tone interaction in non-right-handers (*F*(1,20) = 1.56, *p =* 0.23) and, unlike the theta:alpha categorization no handedness x alertness x side interaction when compared with the right-handed group (*F*(1,41) = 0.46, *p =* 0.50) although it should be noted that 9 participants were absent from this comparison due to no trials on which a response was made being categorized as > Hori 3. Similarly, in the previous report, 19 right-handed participants had valid data for the omission-based analysis. Here, only 13 non-right-handers had valid data and this too indicated no significant interaction between omission defined alertness x side-of-tone interaction (*F*(1,12) = 1.23, *p* = 0.29) and no handedness x alertness x side-of-tone interaction when handedness groups were compared (*F*(1,30) = 1.25, *p* = 0.27).
